# Coping With Adolescents Affected by Anorexia Nervosa: The Role of Parental Personality Traits

**DOI:** 10.3389/fpsyg.2021.678745

**Published:** 2021-07-07

**Authors:** Alessio Maria Monteleone, Alberta Mereu, Giammarco Cascino, Maria Chiara Castiglioni, Chiara Marchetto, Melissa Grasso, Maria Pontillo, Tiziana Pisano, Stefano Vicari, Valeria Zanna

**Affiliations:** ^1^Department of Psychiatry, University of Campania “Luigi Vanvitelli”, Naples, Italy; ^2^Child and Adolescent Psychiatry, Center of Excellence in Neuroscience, Children’s Hospital A. Meyer-University of Florence, Florence, Italy; ^3^Department of Medicine, Surgery and Dentistry ‘Scuola Medica Salernitana’, Section of Neurosciences, University of Salerno, Salerno, Italy; ^4^Child Neuropsychiatry Unit, Department of Neuroscience, I.R.C.C.S. Children Hospital Bambino Gesù, Rome, Italy

**Keywords:** anorexia nervosa, caregiving, personality, coercion, collusion, illness duration

## Abstract

**Introduction:**

Anorexia nervosa (AN) promotes psychological distress in caregivers who adopt different coping strategies. Dysfunctional caregiving styles exacerbate further distress in the patient promoting the maintenance of the illness. We aimed to assess the possible contribution of personality traits of caregivers to the adoption of different coping strategies to deal with the affected relative.

**Methods:**

About 87 adolescents with AN were recruited. Their parents completed the Family Coping Questionnaire for Eating Disorders (FCQ-EDs) and the Temperament and Character Inventory-Revised (TCI-R). Differences between mothers and fathers were assessed through the independent sample *t*-test. Multivariate regression analyses were run to assess if personality traits, the occurrence of psychiatry conditions in the parents, the marital status, and the duration of the illness predicted parental coping strategies.

**Results:**

The group of mothers showed higher levels of avoidance and seeking for information coping strategies than the sample of fathers. Lower illness duration predicted higher collusion with the illness in both parents. Harm avoidance, cooperativeness, and self-directedness positively predicted parental coercion, collusion, and seeking for information strategies with some differences between mothers and fathers.

**Discussion:**

Illness duration and personality traits of parents affect the type of parental coping strategies developed to face AN in adolescents. These variables should be considered in the assessment of families of adolescents with AN and may be addressed to promote more fine-tuned clinical interventions for caregivers.

## Introduction

Anorexia nervosa (AN) is a complex psychiatric illness with a severe impact on physical and social domains (Monteleone et al., 2019; Treasure et al., 2020). Social functioning is reduced both before and after illness onset (Cardi et al., 2018a): people with AN describe their illness with the word “isolation” (McKnight and Boughton, 2009) and show the low quality of relationships with peers before symptoms develop (Cardi et al., 2018b), while a wide range of alterations in social cognitive processes has been found in the acute phase of the illness (Monteleone et al., 2018, 2020) and partially after recovery (Oldershaw et al., 2011).

The social aspects of the illness also include the family network. The family relationships may either contribute to the AN development/maintenance (Schmidt and Treasure, 2006; Treasure and Schmidt, 2013) or are seriously affected by the AN onset (Treasure and Nazar, 2016). Indeed, AN typically starts in adolescence (Volpe et al., 2016) and people with AN often remain dependent upon their families during their lifetime (Hjern et al., 2006). Parents are involved in providing meal support, emotional, and financial help and may be essential to bridge isolation of their affective relative, especially in adolescents (Murray et al., 2015).

However, the parents also experience a wide range of emotional and behavioral reactions to the onset of AN symptoms in their relative and this may have a negative impact on the illness course (Treasure and Nazar, 2016). Dysfunctional parental coping strategies range from critical, hostile, and overcontrolling behaviors to accommodation to the eating disorder (ED) rules and avoidance of the illness (Treasure et al., 2020). According to a recent study (Parks et al., 2018), the mothers of adolescents with EDs adopt self-sufficient problem-focused strategies more than mothers of healthy adolescents and this may result in hostility and overinvolvement. Although further research is a need in this field, some differences in the reactions of parents have been detected between mothers and fathers, the former being more emotional overinvolved, less critical (Anastasiadou et al., 2016), and more prone to search for social support (Parks et al., 2018) than the fathers.

The adoption of maladaptive coping strategies may either be due to Anastasiadou et al. (2014) or contribute to Coomber and King (2012) the high levels of emotional and psychological distress occurring in caregivers of people with EDs. Treasure and Nazar (2016) hypothesized that several factors may promote specific caregiver coping strategies and their burden in the presence of the anorexic symptoms. These factors are clustered together in three domains: illness-related variables (i.e., the illness stage or the severity of symptoms), societal reactions to the illness (i.e., the stigma or the social support perceived by the family), and the variables of caregivers (which refer to their skillful aspects). In this line, Sepúlveda et al. (2012) found that the social isolation of parents, their educational levels, and their fear of the dangers related to physical health predicted their emotional well-being. However, the factors related to caregivers need to be further explored. Indeed, personality characteristics can affect emotional regulation and communication skills and, thus, the use of different behavioral strategies to cope with stressful events. Although clusters of personality traits of parents have been identified as associated with the psychopathology features of some daughters (Fassino et al., 2009; Amianto et al., 2013, 2015), no study has assessed the possible contribution of the personality of parents to the assumption of different coping strategies. This study aims to address this literature gap in adolescents with AN. We also explored differences in coping strategies between mothers and fathers, which have been not sufficiently assessed (Anastasiadou et al., 2014). Given the lack of previous studies, we had no prior hypothesis on the specific association between personality traits and the behaviors of caregivers.

## Materials and Methods

### Participants

Participants in this study were recruited from patients consecutively admitted and hospitalized for their ED to the Child and Adolescent Neuropsychiatry Unit of the Clinical and Research Hospital “Bambino Gesù” of Rome, Italy, and the Child and Adolescent Psychiatry Unit, A. Meyer Children’s Hospital, Florence, Italy, if they met the following inclusion criteria: (a) age ≤ 18 years; (b) current diagnosis of AN or atypical AN according to the DSM-5 criteria and confirmed by the Schedule for Affective Disorders and Schizophrenia for School-Age Children-Present and Lifetime Version (K-SADS-PL) (Kaufman et al., 2016); (c) absence of current and/or lifetime comorbid psychiatric disorders such as substance abuse disorder, schizophrenia, and bipolar disorder; and (d) living with both parents or with one of two if divorced. The diagnostic assessment was made by expert psychiatrists (VZ and AM), who made the diagnosis first through a face-to-face clinical interview and then employing the K-SADS-PL to confirm the AN diagnosis and psychiatric comorbidity. For each patient, the two parents were invited to participate in the study if they were actively involved in the care of the patient. Demographic characteristics (age, marital/living status, and presence of a first degree relative with a current or previous diagnosis of ED) and the presence of psychiatric disorders in the parents of the recruited patients were assessed employing the Structured Clinical Interview for DSM-5 Disorders–Research Version (SCID-RV) (First, 2016). The duration of the illness was evaluated considering the time elapsed between the onset of the symptoms and the moment of hospital care: this data was collected through the diagnostic interview KSADS-PL, administered to both parents and the child.

The final study sample included 87 patients (60 with AN restricting type, 7 with AN binge-purging type, and 20 with atypical AN), 87 mothers, and 87 fathers.

### Procedure and Measures

The study was approved by the Institutional Board of the Child and Adolescent Neuropsychiatry Unit of the Clinical and Research Hospital “Bambino Gesù” of Rome (number: 2288_OPBG_2020) and was carried out in accordance with the Declaration of Helsinki for experiments involving humans. All the participants and their parents gave their written consent after being fully informed of the nature and procedures of the study.

The parents were asked to fill in the following questionnaires before patients entering specific treatment programs: the Italian version of the Family Coping Questionnaire for Eating Disorders (FCQ-EDs) (Fiorillo et al., 2015) and the Temperament and Character Inventory-Revised (TCI-R) (Fossati et al., 2007).

The FCQ-ED questionnaire (Fiorillo et al., 2015) assessed the coping strategies of parents to face the illness of patients. The questionnaire consists of 32-items exploring six domains: collusion with the behaviors of the patient (i.e., not saying anything regarding dysfunctional eating symptoms of the patient); coercion (i.e., angry reactions of parents to behaviors of the patient); avoidance (i.e., parents avoid situations that remind the illness of patient); positive communication with the patient (i.e., calming and reassuring reactions of parents); seeking for information on the illness of patient (i.e., trying to get information on the illness); and seeking for spiritual help (single item). In the original version of the questionnaire (Fiorillo et al., 2015), coercion, collusion, and avoidance subscales clustered in a unique factor describing the emotional coping strategies of parents, while positive communication with the patient and seeking for information on the illness of patient subscales clustered in a factor pointing to problem-solving strategies. For this reason and in accordance with literature evidence (Treasure and Nazar, 2016), we did not include the seeking for spiritual help item in the analyses. Cronbach’s values of each FCQ-ED subscore ranged between 0.65 and 0.87 in the group of fathers and between 0.69 and 0.88 in the group of mothers.

The TCI-R (Fossati et al., 2007) investigates personality characteristics. It is a 5-point Likert-type scale questionnaire grouped in seven subscales assessing four temperament dimensions (i.e., novelty seeking, harm avoidance, reward dependence, and persistence) and three character dimensions (i.e., self-directedness, cooperativeness, and self-transcendence). Temperamental features are considered inheritable and appearing in the first years of life, while character dimensions are developed during life resulting from social experiences (Cloninger et al., 1993). Cronbach’s values of each TCI subscore ranged between 0.68 and 0.81 in the group of fathers and between 0.67 and 0.87 in the group of mothers.

### Statistical Analyses

Differences between mothers and fathers in coping strategies and personality characteristics were investigated by means of the independent sample *t*-test through JASP software JASP Team (2020). Bonferroni corrections for multiple testing were applied dividing 0.05 by the overall number (12) of questionnaire comparisons. The level of significance was set at 0.004.

Multivariate regression analyses were conducted using the lavaan package (Rosseel, 2012) in R, Version 3.6.1 (R core Team, Vienna, Austria). The temperament and character scores of parents, illness duration (less than 6 months, between 6 and 12 months and above 12 months), the marital status (joined or divorced), and the occurrence of psychiatric disorders in the parents were included as predictors and the coping strategies of the parent as dependent variables. The simultaneous effect of each predictor on each dependent variable was evaluated, taking into account the covariance among variables.

These analyses were performed separately for each parent.

## Results

### Patients Characteristics

The age of patients ranged between 11.1 and 18 years (mean: 15.4; SD: 1.5). The mean BMI was 16.5 ± 2.1. No comorbid psychiatric disorder was detected in 40 (45.9%) patients, while a comorbid depressive disorder was revealed in 27 (31%) patients, a comorbid anxiety disorder was diagnosed in 27 (31%) patients, and both anxiety and depressive disorder were observed in nine (10.3%) patients. Four (4.6%) patients were diagnosed with a comorbid obsessive-compulsive disorder. At referral, 22 (25.2%) patients were receiving antidepressant drugs, 22 (25.2%) patients were receiving antipsychotic drugs, and nine (10.3%) patients were receiving benzodiazepine. Illness duration was less than 6 months in 29 (33.3%) patients, ranged from 6 to 12 months in 32 (36.8%) patients, and was longer than 12 months in 26 (29.9%) patients.

### Parents Characteristics

Demographic and clinical characteristics of the group of mothers and fathers are reported in Table 1. About 16 (18.4%) patients had a first-degree relative with a current or previous diagnosis of ED. The parents of 17 patients (19.5%) were divorced. Sixteen (18.4%) mothers and six (6.9%) fathers were diagnosed with a current depressive disorder; 12 (13.8%) mothers and eight (9.2%) fathers were diagnosed with a current anxiety disorder.

**TABLE 1:** Demographic and clinical characteristics of the group of mothers and fathers.

	**Mother**	**Father**	***t***	***p***	**Cohen’s *d***
Age	48.24 ± 4.46	51.38 ± 5.38	4.17	<0.001*	0.64
**TCI-R**					
Novelty seeking	98.53 ± 14.20	97.75 ± 15.07	0.35	0.72	0.05
Harm avoidance	96.67 ± 19.20	90.88 ± 16.02	2.22	0.03	0.32
Reward dependence	107.37 ± 16.10	98.13 ± 14.62	5.04	<0.001*	0.60
Persistence	112.16 ± 18.27	112.68 ± 18.70	0.18	0.85	0.02
Self-directedness	149.15 ± 20.79	146.41 ± 25.41	0.77	0.43	0.11
Cooperativeness	138.95 ± 17.48	133.15 ± 17.28	2.20	0.02	0.33
Self-transcendence	70.54 ± 15.59	67.63 ± 14.05	1.29	0.19	0.19
**FCQ-ED**					
Avoidance	3.26 ± 2.47	2.30 ± 2.39	2.55	0.01	0.39
Coercion	28.33 ± 6.48	26.48 ± 9.55	1.47	0.14	0.22
Collusion	17.64 ± 5.04	18.22 ± 5.71	0.88	0.37	0.13
Information	5.31 ± 1.34	4.87 ± 1.39	2.06	0.04	0.31
Positive communication	29.85 ± 4.64	29.12 ± 7.22	0.79	0.43	0.12

TCI-R, Temperament and Character Inventory-Revised; FCQ-ED, Family Coping Questionnaire for Eating Disorders.

*Significant after Bonferroni correction.

The *t*-test (Table 1) showed that mothers scored higher than fathers on seeking information and avoidance subscales. These differences did not persist after Bonferroni corrections. No differences were detected for the others FCQ-ED subscales between the parents. The *t*-test for the TCI-R subscales displayed that harm avoidance, reward dependence, and cooperativeness were significantly higher in mothers than in fathers, although only differences related to reward dependence persisted after Bonferroni corrections (Table 1).

### Regression Analyses

**FIGURE 1:**
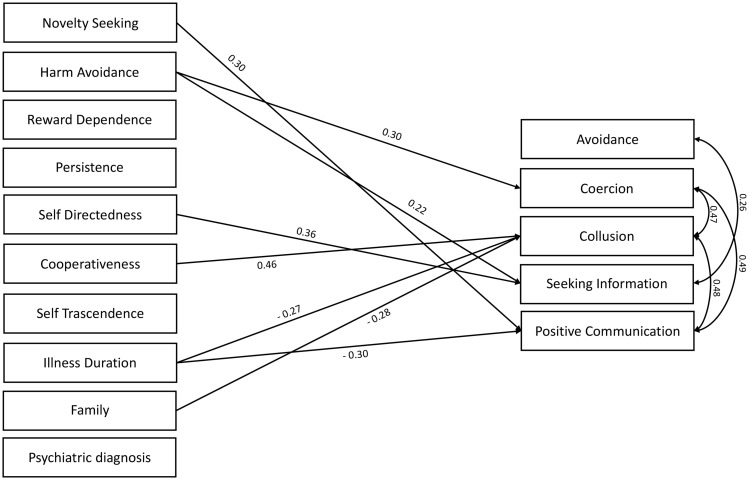
Multivariate regression analyses in the sample of fathers. Standardized beta is reported on the arrows (df = 45).

Results of the multivariate regression analyses in fathers are reported in Figure 1. Coercion was significantly and positively predicted by harm avoidance. Collusion was positively predicted by cooperativeness and negatively predicted by the marital status and by illness duration, indicating that joined families and shorter illness duration predict higher levels of collusion. Avoidance was not significantly predicted by the TCI-R subscores. Positive communication with the patient was positively predicted by novelty seeking and negatively predicted by illness duration. Seeking information on the illness of patient was positively predicted by harm avoidance and self-directedness.

**FIGURE 2:**
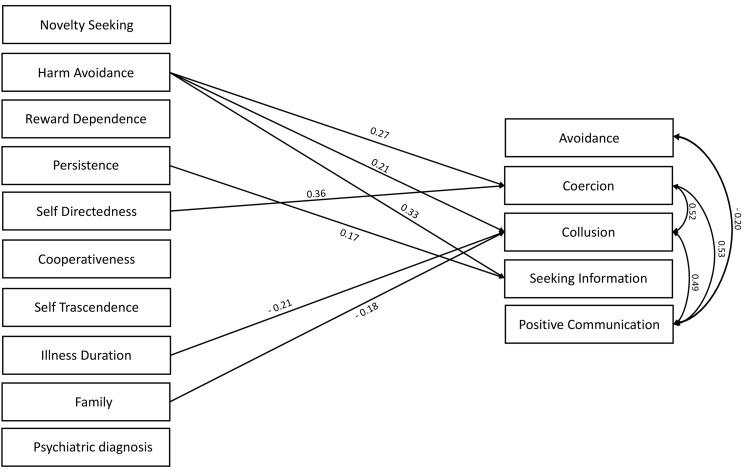
Multivariate regression analyses in the sample of mothers. Standardized beta is reported on the arrows (df = 45).

Results of the multivariate regression analyses in mothers are reported in Figure 2. Harm avoidance and self-directedness were positive predictors of coercion. Collusion was positively predicted by harm avoidance, while it was negatively predicted by the marital status and by illness duration, indicating that joined families and shorter illness duration predict higher levels of collusion. Avoidance and positive communication with the patient were not significantly predicted by the TCI-R subscores. Seeking information on the illness of patient was positively predicted by harm avoidance and persistence.

In both parents, coercion and collusion scores were positively associated with each other and with positive communication. Finally, avoidance was positively associated with seeking information and negatively associated with positive communication in fathers and mothers, respectively.

## Discussion

This study investigated caregiving differences between mothers and fathers and personality-related factors of parents possibly promoting different coping strategies to face AN in adolescents. The group of mothers showed higher levels of seeking information and avoidance strategies than the sample of fathers. In both parents, the collusion was negatively associated with the illness duration and was higher when the parents were joined, while the occurrence of psychiatry disorders in the parents did not affect the adoption of parental coping strategies. The personality traits of parents were significant predictors of their caregiving style: harm avoidance was positively associated with coercion and seeking for information in both parents as well as with collusion in mothers. Some differences were detected between mothers and fathers, given that in the group of fathers, collusion was positively predicted by cooperativeness, while positive communication with the patient was higher at illness onset and positively predicted by novelty seeking; in the group of mothers, instead, coercion was predicted by self-directedness.

The first interesting aspect of this study is the investigation of parental coping strategies in adolescents with a diagnosis of AN. Adolescence is the lifetime period when usually AN starts (Volpe et al., 2016); thus, it is ideal to assess the parental reactions to the illness before further psychosocial impairment occurs (Treasure et al., 2020). Remarkably, family-based treatments are highly recommended in the first phase of the illness (Lock, 2015), highlighting the importance of studying how parents cope with the illness of patients in adolescence. In accordance with this background, we found that illness duration was a significant negative predictor of the collusion of mothers and fathers and a negative predictor of positive communication of fathers with the patients. These findings suggest that at illness onset, both parents tend to accommodate the illness (i.e., by organizing the family around the ED rules) and that fathers have more calming and reassuring reactions than in later stages of the illness. This is consistent with Rhind et al. (2016), who showed high levels of accommodation with the illness in mothers of adolescents with AN, and it adds to the positive association between the avoidance of relatives and illness duration of the patients found in caregivers of adults with AN (Fiorillo et al., 2017). In this line, the age of parents negatively predicted the use of avoidant coping strategies in caregivers of adolescents with EDs (Parks et al., 2018). Although differences between the illness stages (i.e., a comparison between adolescents vs. adults) have not been thoroughly explored in this study, these results support the staging model of AN (Treasure et al., 2015b) highlighting the possibility that also coping mechanisms of parents may differ throughout AN.

To the best of our knowledge, only two studies (Rhind et al., 2016; Parks et al., 2018) assessed gender differences related to the parental coping strategies in adolescents with AN displaying that mothers exhibit higher levels of overprotective and accommodating behaviors (Rhind et al., 2016), search more social support to cope with the illness and detect more positive and negative aspects of their caregiving (Parks et al., 2018) than fathers. Furthermore, the mother’s avoidance was positively predicted by a more severe perception of the illness (Parks et al., 2018). The present study revealed higher avoidance and seeking for information in mothers than in fathers, although these differences did not persist after Bonferroni corrections. Despite different instruments were employed in these studies, the findings are not at odds with the previous ones given that avoidance is a component of the accommodating construct measured in the Rhind et al. (2016) study. Moreover, the emotional expression assessed by Rhind et al. (2016) pointed to criticism and overinvolvement and the lack of difference between the parents found in that study is in line with the lack of difference in terms of coercion shown in this study. In accordance with adult studies pointing to less maladaptive coping strategies in mothers (Fiorillo et al., 2017), we found higher seeking for information (which is an adaptive strategy) also in mothers of adolescents. However, the present findings are in contrast with studies displaying higher maternal emotional involvement and higher paternal criticism in parents of adult people with AN (Kyriacou et al., 2008; Whitney et al., 2012): differences in illness duration among the study samples are likely responsible for such a discrepancy. These results highlight the importance to consider illness duration when assessing gender differences between parents, even in adolescents in the first years of their illness.

A second innovative aspect of the study is that it is the first one showing that the caregiving style of parents to cope with AN is influenced by personality traits and characteristics. Harm avoidance was a positive predictor of coercion and of seeking for information on the illness of patient in both parents. In mothers, harm avoidance also predicted collusion. Harm avoidance implies a predisposition to depressive, anxious, and stressful reactions to stressful events with low tolerance of uncertainty (Cloninger et al., 1993). Harm avoidance is high in fathers of restricting women with AN (Fassino et al., 2009), it was included in the most suffering personality clusters of mothers and fathers of people with EDs (Amianto et al., 2013), and is a transdiagnostic personality trait of people with EDs (Krug et al., 2011). The present findings show that higher harm avoidance predicts less adaptive emotional behaviors (coercion in both the parents and collusion in the mothers), and suggest that harm avoidance may be an important personality factor to handle the emotional burden triggered by the illness. However, in this analysis, harm avoidance resulted associated also with seeking information about the illness and, thus, it may simultaneously promote different coping strategies. Differences in the association between personality traits and coping strategies between mothers and fathers also emerged. Indeed, across dysfunctional coping strategies, collusion was predicted by cooperativeness in fathers, while coercion was predicted by self-directedness in mothers. These relationships may be comprehensible, given that more tolerant, supportive, and empathic characteristics of people with high cooperativeness (Cloninger et al., 1993) may be associated with higher collusion of fathers with ED behaviors and the ability to orient behaviors according to the goals of an individual (the self-directedness) may promote angry reactions of mothers to the behaviors of the patient. It is worth noting that the presence of psychiatric disorders in the parents had no significant effect on the assessed coping strategies; thus, emotional and behavioral skills connected to personality traits, beyond the occurrence of any psychiatry disorder, may orient their caregiving strategies.

The main strengths of this study are the investigation of parental coping strategies in a sample of adolescents with AN in their first years of the illness and the evaluation of differences between parents. Moreover, no study has previously assessed the contribution of personality traits to orient caregiving strategies in parents of adolescents with AN.

Limitations of the study also need to be acknowledged. First, societal aspects (i.e., social support) that may affect parental caregiving have been not assessed. Second, the family functioning dynamics in terms of the influence of personality traits of each parent or coping strategy on the counterpart have not been included as a possible predicting factor. Third, the size of the sample of patients with atypical AN is not adequate to investigate differences with adolescents with full AN diagnosis.

## Conclusion

The ability of mothers to cope with AN in adolescence seems to be characterized by a trend toward higher avoidance strategies and seeking information with respect to the fathers. In both parents, collusion with the illness is higher at the beginning of the illness. Harm avoidance, self-directedness, and cooperativeness are the personality traits and characteristics that predict maladaptive emotional coping strategies (coercion and collusion) in the parents, with some differences between mothers and fathers. According to literature data (Treasure et al., 2009), distress of parents and the caregiving strategies may promote further distress in the adolescents with AN, constituting a vicious circle. In this line, the “New Maudsley Collaborative Care Approach” showed that caregiving behaviors are associated with the illness outcome (Treasure et al., 2015a). Thus, identifying and addressing the possible determinants of maladaptive parental coping strategies may be essential to improve clinical interventions recently developed to help families with prolonged illness (Cardi et al., 2017) or with adolescents patients with AN (i.e., an adaptation of the acceptance and commitment therapy) (Timko et al., 2015). According to the findings of the present study, clinicians are advised to consider that the reactions of parents to the illness of children may be associated with their emotional and interpersonal skills, as highlighted by their personality traits, and illness duration. Taking into account these personality traits may allow clinicians to provide a fine-tuned and grained approach to the families of adolescents with AN.

## Data Availability Statement

The raw data supporting the conclusions of this article will be made available by the authors, without undue reservation.

## Ethics Statement

The studies involving human participants were reviewed and approved by the Institutional Board of the Child and Adolescent Neuropsychiatry Unit of the Clinical and Research Hospital “Bambino Gesù” of Rome. Written informed consent to participate in this study was provided by the participants’ legal guardian/next of kin.

## Author Contributions

AMM and VZ: conceptualization. MC, CM, MG, MP, and AM: data curation and investigation. GC: formal analysis. AMM, AM, VZ, and GC: methodology. VZ, TP, and SV: project administration. AMM: roles/writing the original draft. AMM, AM, and VZ: writing the review and editing. All authors contributed to the article and approved the submitted version.

## Conflict of Interest

The authors declare that the research was conducted in the absence of any commercial or financial relationships that could be construed as a potential conflict of interest.
